# BDNF promotes mouse follicular development and reverses ovarian aging by promoting cell proliferation

**DOI:** 10.1186/s13048-023-01163-9

**Published:** 2023-04-27

**Authors:** Bin Liu, Yongjie Liu, Shuman Li, Pingping Chen, Jun Zhang, Liping Feng

**Affiliations:** 1 Ministry of Education-Shanghai Key Laboratory of Children’s Environmental Health, Xinhua Hospital, School of Medicine, Shanghai Jiao Tong University, Shanghai, China; 2 Department of Reproduction, Xinhua Hospital, School of Medicine, Shanghai Jiao-Tong University, Shanghai, China; 3 Department of Obstetrics and Gynaecology, Duke University, Durham, NC USA

**Keywords:** BDNF, Aging, Ovarian, Follicles, Oocyte, Creb, cyclinD1, trkB

## Abstract

**Background:**

Brain-derived neurotrophic factor (BDNF) plays an important role in ovarian function including follicle development and oocyte maturation, and embryonic development. However, whether BDNF treatment can reimpose ovarian aging and impaired fertility remains elusive. In this study, we investigated the reproductive outcomes of BDNF treatment and potential mechanisms in aged mice.

**Method:**

“Aged” mice (35–37 weeks old, n = 68) were treated with recombinant human BDNF protein (rhBDNF, 1 µg/200 µL) through daily intraperitoneal (IP) injection for 10 days with/without ovulation induction. Reproductive age mice (8–10 weeks old, n = 28) were treated with ANA 12 (a selective BDNF receptor, TrkB antagonist) through daily IP injection for 5 days with/without ovulation induction. Ovarian function was assessed by ovarian weight, number of follicles, and sex hormone productions. Following induction of ovulation, the number of total oocytes or abnormal oocytes, and blastocyst formation were assessed. Reproductive functions of the mice were evaluated, including pregnancy rate, mating duration for conception, implantation sites, litter size, and weight of offspring. Finally, the molecular mechanism of the effects of BDNF on ovarian cell functions in mice were examined by Western blot and immunofluorescence.

**Results:**

rhBDNF treatment increased the ovarian weight, number of follicles, number and quality of oocytes including increased blastocysts formation, blood estrogen levels, and pregnancy rate in 35-37-week-old mice. Conversely, BDNF receptor antagonist, ANA 12, treatment decreased the ovarian volume and number of antral follicles and increased the proportion of abnormal oocytes in 8-10-week-old mice. We further demonstrated that BDNF treatment promoted ovarian cell proliferation as well as activation of TrkB and cyclinD1-creb signalling.

**Conclusion:**

We demonstrated that ten consecutive days of daily IP injection of rhBDNF rescued ovarian function in aged mice. Our results further indicate that TrkB and cyclin D1-creb signaling may underlie the BDNF function in ovaries. Targeting BDNF-TrkB signaling is a potential novel therapeutic strategy to reverse ovarian aging.

**Supplementary Information:**

The online version contains supplementary material available at 10.1186/s13048-023-01163-9.

## Introduction

As the human life expectancy increases, the number of aging-related diseases has significantly increased, and the aging and functional decline of various organs can cause serious disorders [1]. The ovary ages faster than other body systems [2], and this leads to decreased fertility and endocrine-related diseases [3]. Worldwide, 11% of women give birth for the first time over the age of 35, and this percentage rises every year [1, 4]. Additionally, 1–5% of women under age 40 suffer from premature ovarian failure [5, 6]. Ovarian aging is one of the major risk factors for female infertility, which affects all aspects of people’s lives including psychological issues and reduced quality of life.

Ovarian senescence is characterized by a gradual decline in the number and quality of follicles [2], but the mechanism remains elusive. Sinus follicle count has been used as an indicator for assessing ovarian reserve [7]. In animals, the ovarian reserve usually reflects the number of follicles at various stages, reproductive capacity, and endocrine function. Therefore, delaying ovarian senescence is of great significance for improving female fertility and preventing endocrine-related diseases. Free radicals and oxidative stress have been shown to accelerate aging of the ovaries and reduce their function [8–11]. In recent years, scientists have aimed to improve ovarian function by reducing oxidative stress and the subsequent damage using antioxidants. Antioxidants such as vitamins C and E [12], N-acetyl-L-cysteine [13], curcumin [14], Coenzyme Q10 [15], and anthocyanin [16] have been shown to improve ovarian function, but their effects are limited and they only mildly delay ovarian aging. Considering the narrow reproductive window for fertility, innovative, fast-acting therapeutic strategies are needed to improve ovarian function and even reverse ovarian aging [17].

In a recent decade, researchers discovered that Brain-Derived Neurotrophic Factor (BDNF) plays an important role in ovarian functions. The main function of BDNF is to promote the growth and differentiation of neurons and maintain their function [18, 19]. BDNF plays important roles in other tissues, such as promotion of blood vessel formation [20], cell proliferation, and adhesion, and has anti-apoptotic effects and inhibits tumor metastasis [21–24]. Strikingly, BDNF also plays a significant role in the reproductive system. BDNF has been found in granulosa cells, oocytes, and embryos [20, 21, 25]. Studies have found that the addition of exogenous BDNF to embryo cultures increased the quality of embryos and the rates of fertilization and blastocyst formation [22, 23]. Treatment with exogenous BDNF also significantly improved the maturation rate of oocytes in immature oocyte cultures [26]. BDNF can promote follicle development, oocytes maturation in ovaries, and embryo development and blastocyst formation in vitro [27, 28]. Tropomyosin receptor kinase B (TrkB) is the main receptor of BDNF and mediates the BDNF signaling in follicular development [29, 30].Taken together, BDNF plays an important role in ovarian function.

However, whether targeting BDNF-TrkB signaling can reimpose ovarian function in aged ovaries is largely unknown. Recently, a TrkB agonist antibody ameliorated fertility deficits in aged and cyclophosphamide-induced premature ovarian failure mouse models [31]. In this study, we investigated the reproductive outcomes, including ovary function and pregnancy outcomes, of BDNF treatment in naturally aged mice that represent more common public health issues in women at present. Further, we studied the effects of treatment with ANA 12, a TrkB antagonist, on ovarian function in young mice to explore BDNF-mediated signaling.

## Methods

### Chemicals and reagents

Recombinant human BDNF protein (rhBDNF, cat#: ab206642) was purchased from ABclonal. Product purity is > 95%. Lyophilized rhBDNF protein was reconstituted at 5 µg/mL in 0.9% normal saline. A TrkB antagonist, ANA-12 (MedChemExpress, cat#: HY-12,497), was dissolved at 50 µg/mL in 17% dimethyl sulfoxide (DMSO) that was made in phosphate-buffered saline (PBS, pH 7.4).

### Animals

Female C57BL/6 mice aged 8–10 weeks or 35–37 weeks (Shanghai Sippe-Bk Lab Animal Co., Ltd.) were used in this study. Mice were housed in a specific pathogen-free (SPF) and climate-controlled facility at constant room temperature (22–25 °C) under a 12 h light: 12 h dark cycle, with humidity control (60 ± 10%). Mice were provided with basic mouse chow and distilled water ad libitum at Shanghai Jiao-Tong University School of Medicine, Xinhua Hospital Laboratory Animal Center. Animal care practices were followed by the ethical guidelines of the International Association for the study of Pain regarding the use of laboratory animals and were reviewed and approved by the Animal Committee of Shanghai Jiao-Tong University School of Medicine, Xinhua Hospital Laboratory Animal Centre, Shanghai, China.

### Drug administration

For the experiments in aged mice (35–37 weeks), female C57BL/6 mice were divided into two groups randomly. One group of mice was treated with rhBDNF (1 µg/200 µL) by daily intraperitoneal (IP) injection for 10 days [32, 33]. In parallel, another group of mice was treated with 0.9% normal saline (a vehicle control, 200 µL) by daily IP injection for 10 days [32, 33]. Similarly, for the experiments in normal reproductive age mice (8–10 weeks-old), female C57BL/6 mice were divided into two groups randomly. One group of mice was treated with ANA 12 (0.5 mg/kg), which can block BDNF-TrkB signal [34, 35], by daily IP injection for 5 days. Another group of mice was treated with 17% DMSO (a vehicle control, ~ 200 µL) by daily IP injection for 5 days.

### Ovulation induction, oocyte collection, and embryo cultures

During treatments, oocyte superovulation was induced by an IP injection of Pregnant Mare Serum Gonadotrophin (PMSG, 10 IU) for 48 h followed by an IP injection of hCG (10 IU) for 14 h. Mice were then sacrificed by cervical dislocation, and oviducts were removed. Oocyte-cumulus complexes were collected from the oviduct ampulla and digested in 80 IU/mL hyaluronidase solution (IrvineScientific, cat#: 90,101) before counting oocytes. Mouse oocytes were classified into normal and abnormal according to their morphology. Abnormal oocytes are oocytes with intracytoplasmic abnormalities (dark or granular cytoplasm and cytoplasmic degeneration) or extracytoplasmic abnormalities (distorted zona and abnormal cell division), or shape abnormalities. Sperm were isolated from 8-10-week-old mice and purified in an IVF reagent (Vitrolife, cat#:10,136). After the sperm and oocyte were fertilized in the IVF reagent for 4 h, the fertilized eggs were then transferred to G1 reagent (Vitrolife, cat#:10,128) culture medium for embryo cultures. The embryos were examined under an inverted microscope every 12 h for 5 days.

### Serum collection

Under isoflurane anesthesia, blood samples were collected from the auricula dextra of all mice into 1.5 mL microcentrifuge tubes and kept on ice for 2 h for clotting. Serum was obtained via centrifugation at 3500 × rpm for 15 min and stored at -80 °C for further analysis.

### Histological staining

Ovaries were collected from all mice, fixed in 4% paraformaldehyde (PFA) for at least 24 h at room temperature, and paraffin embedded. Ovaries were serially sectioned into five 5 μm thick sections that were stained with H&E and counted. A primordial follicle was defined as an oocyte surrounded by a single layer of flattened squamous granulosa cells; a primary follicle was an oocyte surrounded by a single layer of cuboidal granulosa cells; a secondary follicle was an oocyte surrounded by two or more layers of cuboidal granulosa cells with no visible antrum; an antral follicle was defined as an oocyte surrounded by several layers of granulosa cells with an antral space. The number of follicles in each stage was presented as a total number of follicles counted in all five Sects. [36–38].

### Immunofluorescence (IF) staining

Ovaries were collected from all mice, fixed in 4% PFA for at least 24 h, and placed in a 20% sucrose solution for at least 24 h and then a 30% sucrose solution for at least 24 h. The ovarian tissues were then made into serial frozen sections of 8 μm thickness. Sections were blocked with a blocking buffer (5% goat serum, 0.3% Triton X-100 in 0.1 M PBS) for 1 h at room temperature and incubated with primary antibody overnight at 4 °C. After three washes with PBS, sections were incubated with the secondary antibodies at 37 °C for 1 h. After washing, DAPI was added to dye the nuclei. Finally, slides were mounted with a mounting medium (R&D, Cat#: 4866-20) and examined with an Olympus inverted fluorescence microscope. The primary antibodies used for IF: Rabbit anti-BDNF (1:200 dilution, Merck, Cat#: SAB2108004), Rabbit anti-Cyclin D1 (1:400 dilution, Cell Signaling Technology, Cat#: 55506s), Rabbit anti-Ki67 (1:500 dilution, Servicebio Biological, Cat#: GB111141) and Rabbit anti-PCNA (1:200 dilution, Servicebio Biological, Cat#: GB13010-1).

### ELISA for serum analysis

The serum levels of mouse estradiol (E2), BDNF, and progesterone (P4) were assessed using enzyme-linked immunosorbent assay (ELISA) kits following manufacturer’s instructions. BDNF protein levels were quantified using Mouse BDNF ELISA Kit (MULTI SCIENCES, Cat#: EK2127-01). The serum levels of E2 were determined by a mouse estradiol, E2 ELISA kit (SAB, Cat#: EK0328). The serum levels of P4 were determined by mouse progesterone, PROG ELISA Kit (SAB, Cat#: EK11173). The optical density (OD) of ELISA results was measured by a microplate reader (Thermo, Multiskan Go1510) at 450 nm, and the concentrations were calculated using the standard curve.

### Western blot

Ovaries were incubated on ice with lysis buffer (Beyotime, Cat#: P0013C) supplemented with PMSF, a proteinase inhibitor. After homogenization, the lysates were centrifuged at 14,000 rpm at 4 °C for 5 min. The supernatant was collected and stored at -80 °C. Protein concentrations of the supernatants were measured using BCA protein assay (Beyotime, Cat#: P0011). The protein samples were denatured and separated on 12% SDS-PAGE gels and then transferred to PVDF membranes (Millipore) by electrical transfer. PVDF membranes were then blocked using Protein Free Rapid Blocking Buffer (Beyotime, Cat#: P0575) for 15 min at room temperature and then incubated overnight at 4 °C with primary antibodies: anti-creb antibody (1:500 dilution, Abcam, Cat#: ab32515), anti-creb (phosphor S133) antibody (1:1000 dilution, Abcam, Cat#: ab254107), anti-cyclinD1 antibody (1:1000 dilution, Cell Signaling Technology, Cat#: 55,506 S), anti-trkb antibody (1:1000 dilution, Abcam, Cat#: ab187041), cleaved caspase-3 (Asp175) antibody (1:1000 dilution, Cell Signaling Technology, Cat#:9661 S) and anti-B-cell lymphoma-2 (bcl2) (1:5000 dilution, Abcam, Cat#: ab182858).

### Reproductivity assessment

After one week of acclimation, female mice (35–37 weeks old) were mated overnight with healthy male mice (8–10 weeks old) at a 1:1 ratio. The presence of a vaginal plug was designated as embryonic day 0.5 (E0.5). For mice that gave birth naturally, we calculated the days of caging until pregnancy and pregnancy rate and recorded the litter size and the pup weight.

### Statistical analysis

All results are given as mean ± SD. Data were assessed using GraphPad Prism 8.0.2 (La Jolla, CA) and SPSS 26.0 software (SPSS, Inc., Chicago, IL, USA). Statistical analysis was performed using an unpaired Student’s t-test for two comparisons or one-way ANOVA for multiple comparisons. A p-value less than 0.05 was statistically significant.

## Results

### rhBDNF treatment increased the volume and weight of ovaries in aged mice

**Fig. 1:**
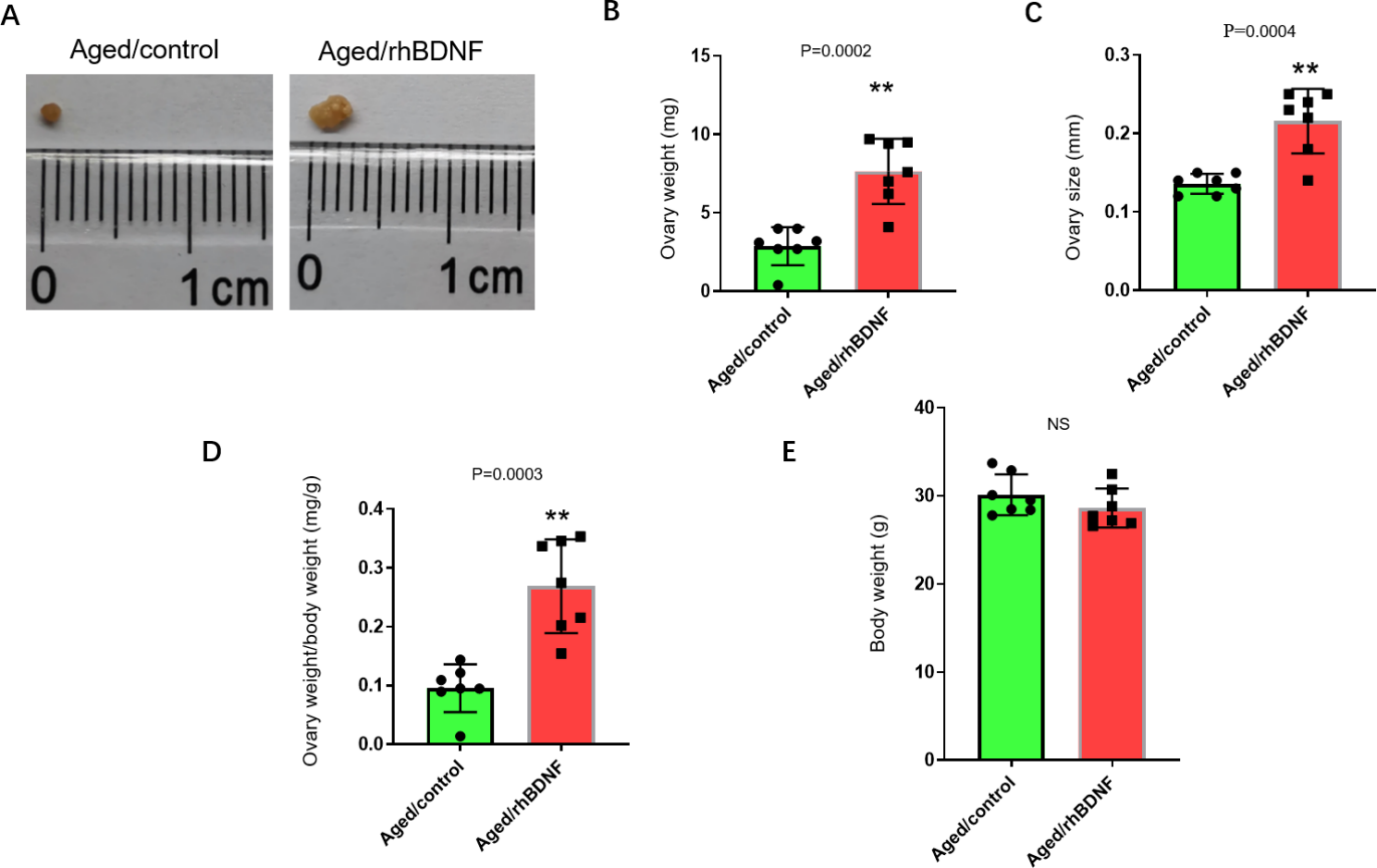
The effects of rhBDNF treatment on the volume (size) and mass (weight) of ovaries in aged mice. (**A**) Ovarian size and appearance. (**B**) Ovarian weight. (**C**) Ovarian size. (**D**) Ovary/body weight ratio. (**E**) Body weight. (n = 7). * indicates P < 0.05, ** indicates P < 0.01. Control is normal saline. Scatter plot data are shown as mean ± SD

The peripheral blood levels of BDNF in 35-37-week-old (aged) mice were significantly decreased compared to reproductive age mice at 8-10-week-old, reproductive-age (young) mice (639.7 vs. 343.8, p<0.01, Fig. S1). Supplementation with rhBDNF (1 µg/day) by daily IP injection for 10 days significantly increased the volume and mass of ovaries compared to the control group (Fig. 1A). Ovarian weight, ovarian size and ovary/body weight ratio were significantly higher in aged mice treated with rhBDNF compared to young mice (Fig. 1B-D), but there were no significant differences in body weight (Fig. 1E).

### rhBDNF treatment improved ovarian function

**Fig. 2:**
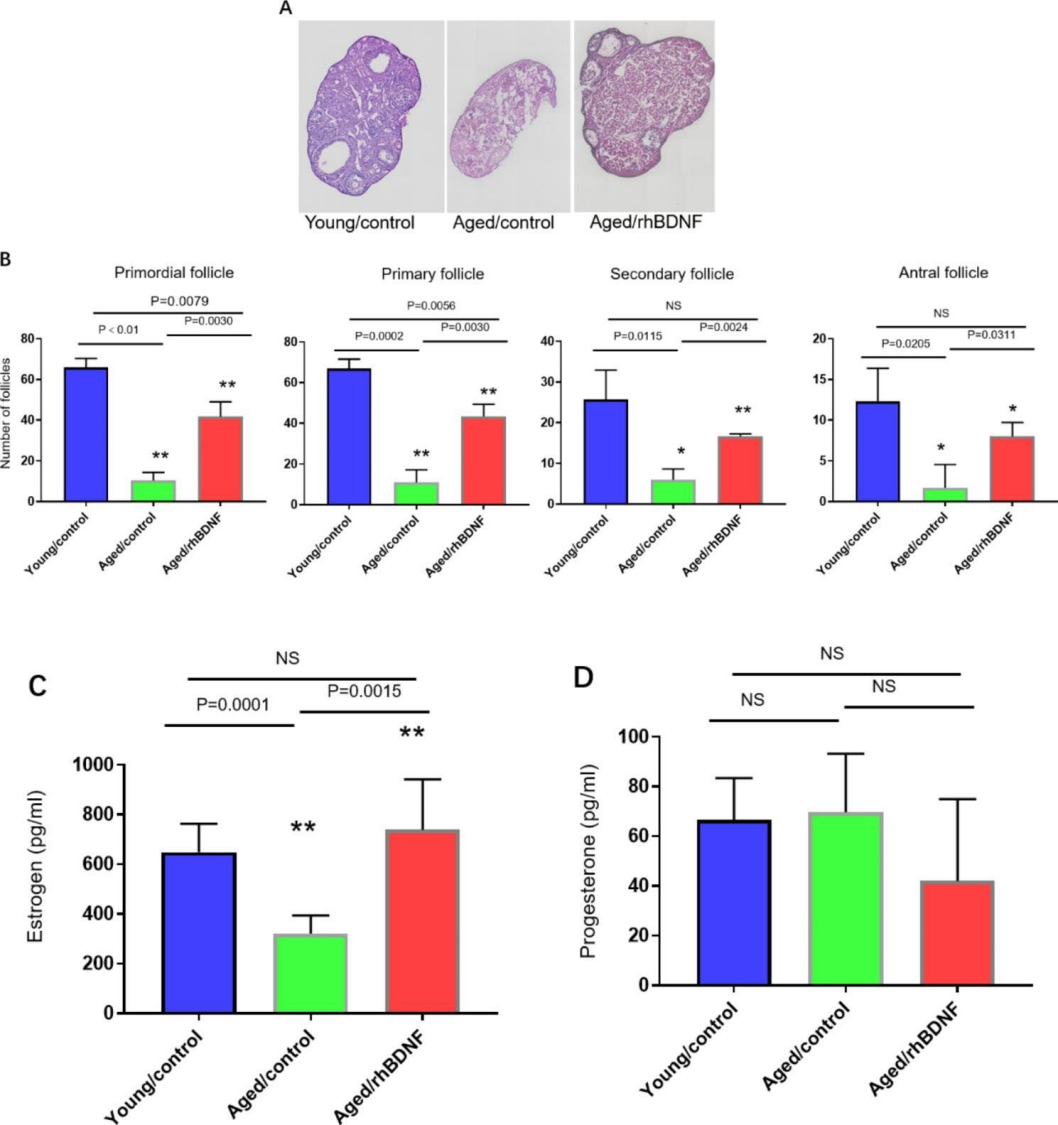
The impacts of rhBDNF treatment on ovarian functions in aged mice. (**A**) H&E staining of ovaries. (**B**) The number of primordial, primary, second, and antral follicles (n = 3). (**C**) Serum estradiol (E2) levels (young/control, n = 8; aged/control, n = 5; aged/rhBDNF, n = 7). (**D**) Serum progesterone (P4) levels (n = 4). Data are shown as mean ± SD. * indicates P < 0.05, ** indicates P < 0.01

The ovaries have two main reproductive functions: producing both oocytes (eggs) for fertilization and the reproductive hormones estrogen, and progesterone. The number of follicles is a predictor of oocyte production. H&E staining was used to examine the ovarian follicle development (Fig. 2A). The number of ovarian follicles in aged mice (35-37-week-old) was significantly reduced compared to young mice (8-10-week-old) (Fig. 2B). rhBDNF treatment significantly increased the number of primordial, primary, secondary, and antral follicles compared to aged mice treated with normal saline (Fig. 2B). The numbers of primordial and primary follicles in rhBDNF-treated aged mice were lower than those in young mice, but the numbers of secondary and antral follicles were not different (Fig. 2B). rhBDNF treatment also significantly increased serum estradiol (E2) levels but not progesterone (P4) levels in aged mice compared to aged mice treated with normal saline (Fig. 2C and D).

### rhBDNF treatment enhanced the number and quality of oocytes in ovulation induction in aged mice

**Fig. 3:**
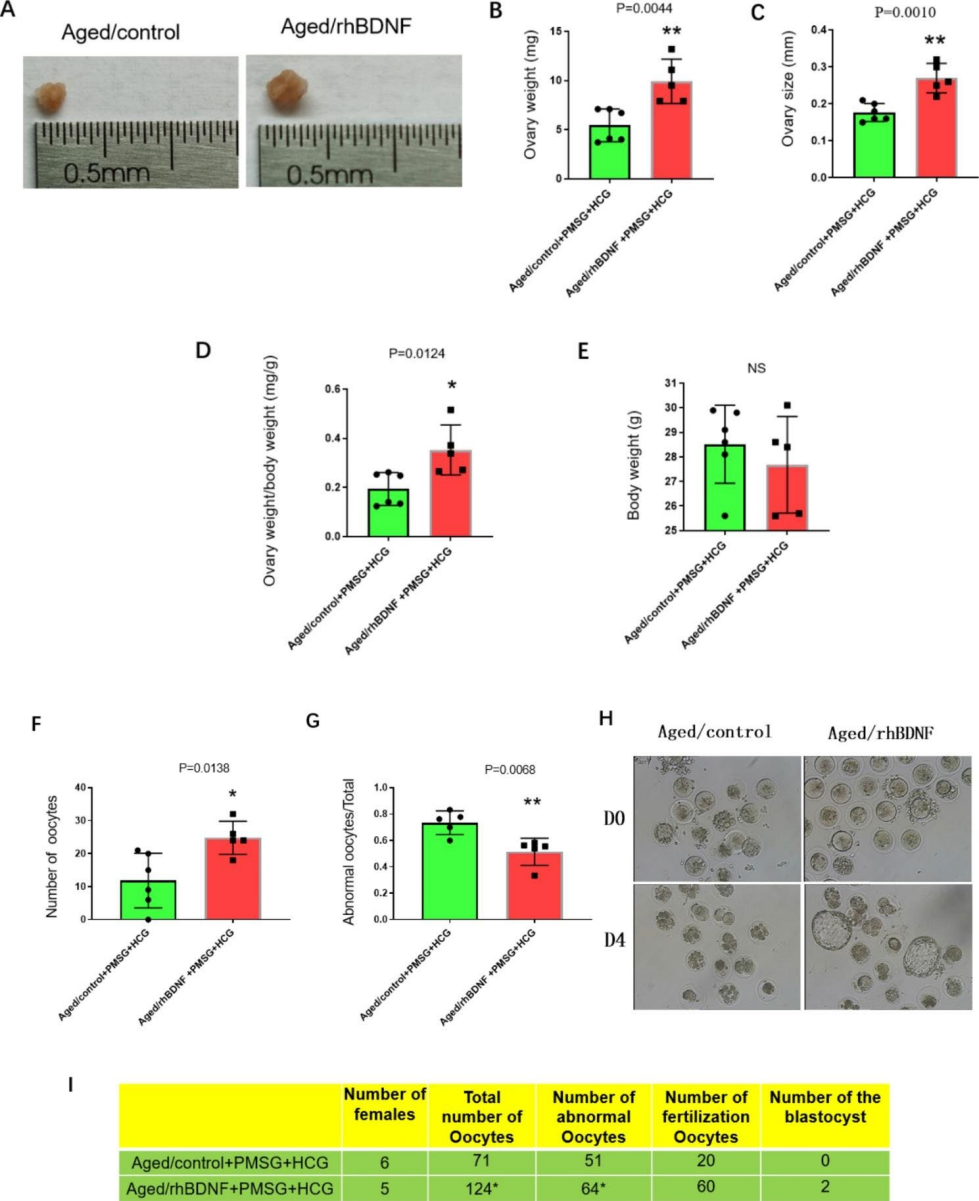
The impacts of rhBDNF treatment on ovulation induction in aged mice. (**A**) Ovarian mass and volume. (**B**) Ovarian weight. (**C**) Ovarian size. (**D**) Ovarian/body weight ratio. (**E**) Body weight. (**F**) The total number of oocytes. (**G**) The ratio of abnormal oocytes to total oocytes. (**H**) Images of oocytes and blastocysts. (**I**) A table summarizing the numbers of mice, total oocytes, abnormal oocytes, fertilized oocytes, and blastocysts. rhBDNF group: n = 5; control group: n = 6. Scatter plot data are shown as mean ± SD. * indicates P < 0.05, ** indicates P < 0.01

We tested the effects of rhBDNF treatment on ovulation induction in aged mice. While undergoing treatment with rhBDNF as described above, the aged mice were treated on day 8 with PMSG (10 IU; IP, 48 h) followed by hCG (10 IU; IP, 14 h) to induce ovulation. The same procedure was performed in the control aged mice treated with normal saline. Ovarian mass and volume (Fig. 3A), ovarian weight and ovarian size (Fig. 3B-C), and ovarian/body weight ratio (Fig. 3D) were significantly increased in the rhBDNF-treated mice compared with the control. There were no differences in body weight (Fig. 3E). The number of oocytes released from the ovaries was significantly increased (Fig. 3F) and the number of abnormal oocytes significantly decreased (Fig. 3G) in the rhBDNF-treated group compared to the control group. We then performed In Vitro Fertilization (IVF) to further determine the quality of the oocytes. The fertilized oocytes were cultured in vitro for 4 days. On the fourth day of embryo cultures, blastocysts were counted. The number of blastocysts was significantly higher in the rhBNDF-treated group compared to the control mice (Fig. 3H). The number of mice, oocytes, abnormal oocytes, fertilized oocytes, blastocytes are shown in Fig. 3I.

### rhBDNF treatment improved the pregnancy rate in aged mice

**Fig. 4:**
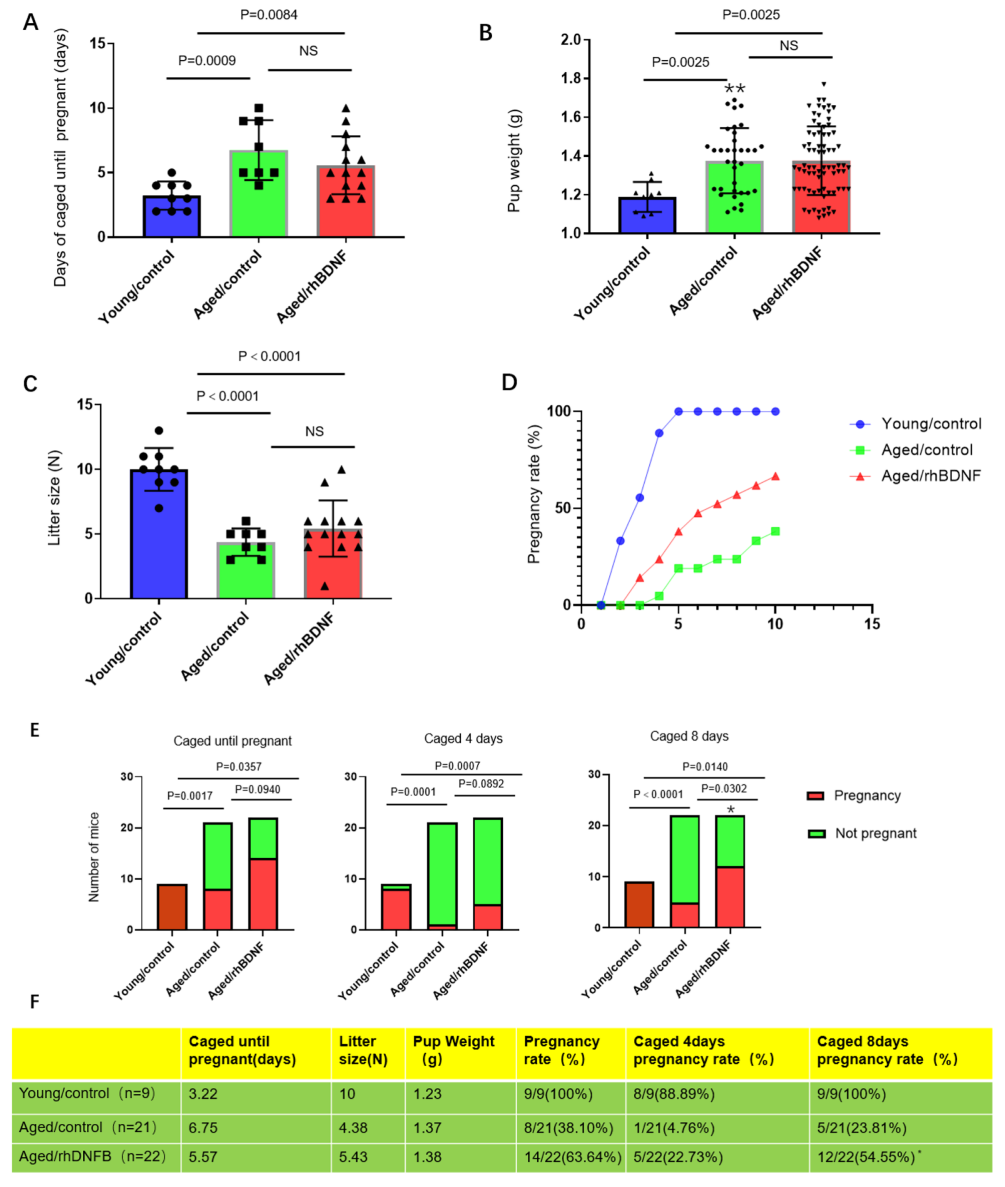
The impact of rhBDNF treatment on fertility in aged mice. (**A**) Days of caging until pregnancy. (**B**) Pup weight. (**C**) Litter size. (**D**) Pregnancy rates. (**E**) The number of mice caged until pregnancy at the 4th day or 8th or 12th day. (**F**) A table summarizing the days of caging until pregnancy, pup weight, litter size, and pregnancy rates. Data are presented as mean ± SD, * indicates P < 0.05, ** indicates P < 0.01

On the 7th day rhBDNF treatment (1 µg/ 200 µL), aged or young mice were mated with 8-10-week-old male mice at a 1:1 ratio. The aged or young female and young male mice were kept in a same cage for 12 days. Reproductive function was assessed using factors including days of caging until pregnancy, pregnancy rate, litter size, and pup weight at birth. Litter size and pup weight at birth were significantly lower in the aged mice compared to the young mice. However, rhBDNF treatment did not significantly alter the days of caging until pregnancy, litter size, and pup weight at birth (Fig. 4A -C). The pregnancy rate was significantly lower in the aged mice compared to young mice. The pregnancy rate on the 8th caged day was significantly increased in rhBDNF-treated mice compared to the control aged mice, but there were no significant differences on other caged days (Fig. 4D and E). The number of days of caging until pregnancy was significantly higher for aged mice vs. young mice, indicating that it took longer time for the aged mice to conceive. These data are summarized in Fig. 4F. The litter size can be determined by a combination of the fertility rate of oocytes, implantation, and fetal development. Thus, we examined the number of embryo implantation sites. On the 7th day of rhBDNF treatment (1 µg/ 200 µL), aged mice were mated with 8-10-week-old male mice at a 1:1 ratio. The aged female and young male mice were kept in a same cage until vaginal plug was observed (gestational day 1). The female mice were sacrificed at day 10.5–11.5 of gestation. The number of embryo implantation sites in rhBDNF-treated aged mice compared to the control aged mice treated with normal saline was not significantly different (Fig. S2A-C). These results suggest that ovarian function, but not the uterus, was the main contributor to the improved reproductive function in aged mice treated with rhBDNF.

### ANA 12 treatment induced the phenotype of ovarian aging in young mice (8-10-weeks-old)

**Fig. 5:**
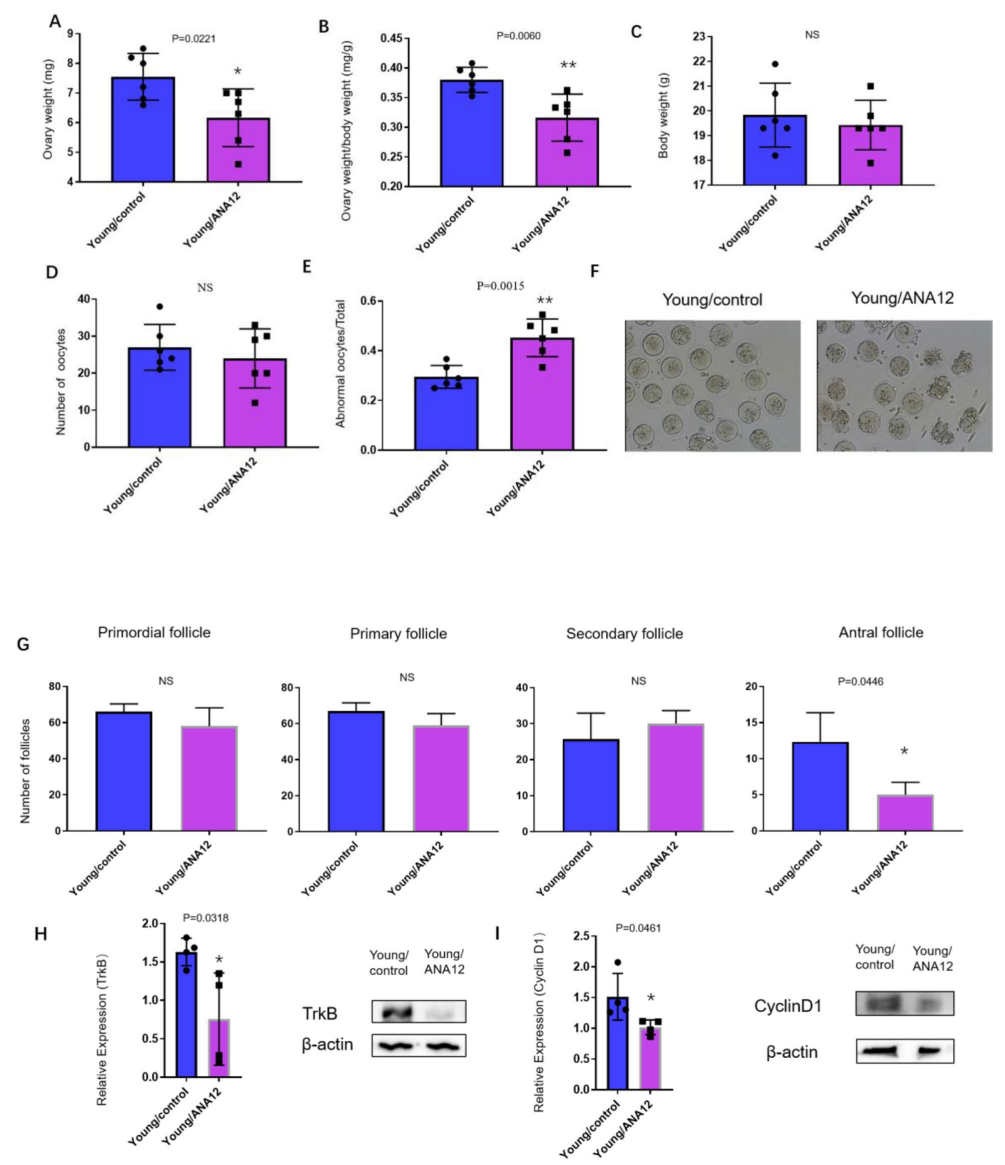
Ovarian function of 8-10-week-old mice treated with ANA 12. (**A**) Ovarian weight. (**B**) The ratio of ovary/body weight. (**C**) Body weight. (**D**) The total number of ovulated oocytes. (**E**) The proportion of abnormal oocytes to total oocytes. (**F**) The images of ovulated oocytes. (n = 6) (**G**) The number of primordial, primary, secondary, and antral follicles. (n = 3). (**H**) The densitometry of TrkB bands in Western blot analysis and a representative Western blot image. (**I**) The densitometry of cyclinD1 bands in Western blot analysis and a representative Western blot image. Data are presented as mean ± SD. * indicates P < 0.05, ** indicates P < 0.01

ANA 12 is a selective, small-molecule non-competitive antagonist of TrkB [39]. Our pilot study determined that 10 days of ANA 12 (0.5 mg/kg) treatment by daily IP injection did not significantly affect the ovarian size and volume compared with 5 days of ANA 12 (0.5 mg/kg) treatment by daily IP injection. The treatment with ANA 12 for 10 days was lethal for some mice, but 5 days of ANA 12 treatment did not cause death or abnormal vital signs in those mice (Fig. S3). Therefore, 5-day treatment was chosen for experiments using ANA 12. TrkB and cyclinD1 protein expression was significantly downregulated in the ANA 12-treated 8-10-week mice for five days compared to the control mice treated with DMSO (Fig. 5H and I). Ovulation was induced on the 3rd day of 5-day ANA 12 treatment (0.5 mg/kg, IP) using PMSG and hCG treatments as described above. Compared to the control mice treated with DMSO, the ovarian weight and the ovarian/body weight ratio were significantly reduced in the ANA 12 treated group (Fig. 5A and B). The body weight and the total number of oocytes released from ovaries were not significantly different between groups (Fig. 5C and D). However, the proportion of abnormal oocytes to total oocytes was significantly higher in the ANA 12-treated mice compared to the control mice treated with DMSO (Fig. 5E and F). H&E staining was used to examine ovarian follicle development. There were no differences in the numbers of primordial, primary, and secondary follicles between groups (Fig. 5G). However, the number of antral follicles was remarkably reduced in the ANA 12-treated mice compared to the control mice (Fig. 5G).

### BDNF promoted ovarian cell proliferation and activated TrkB and cyclin D1-CREB signaling in aged ovaries

**Fig. 6:**
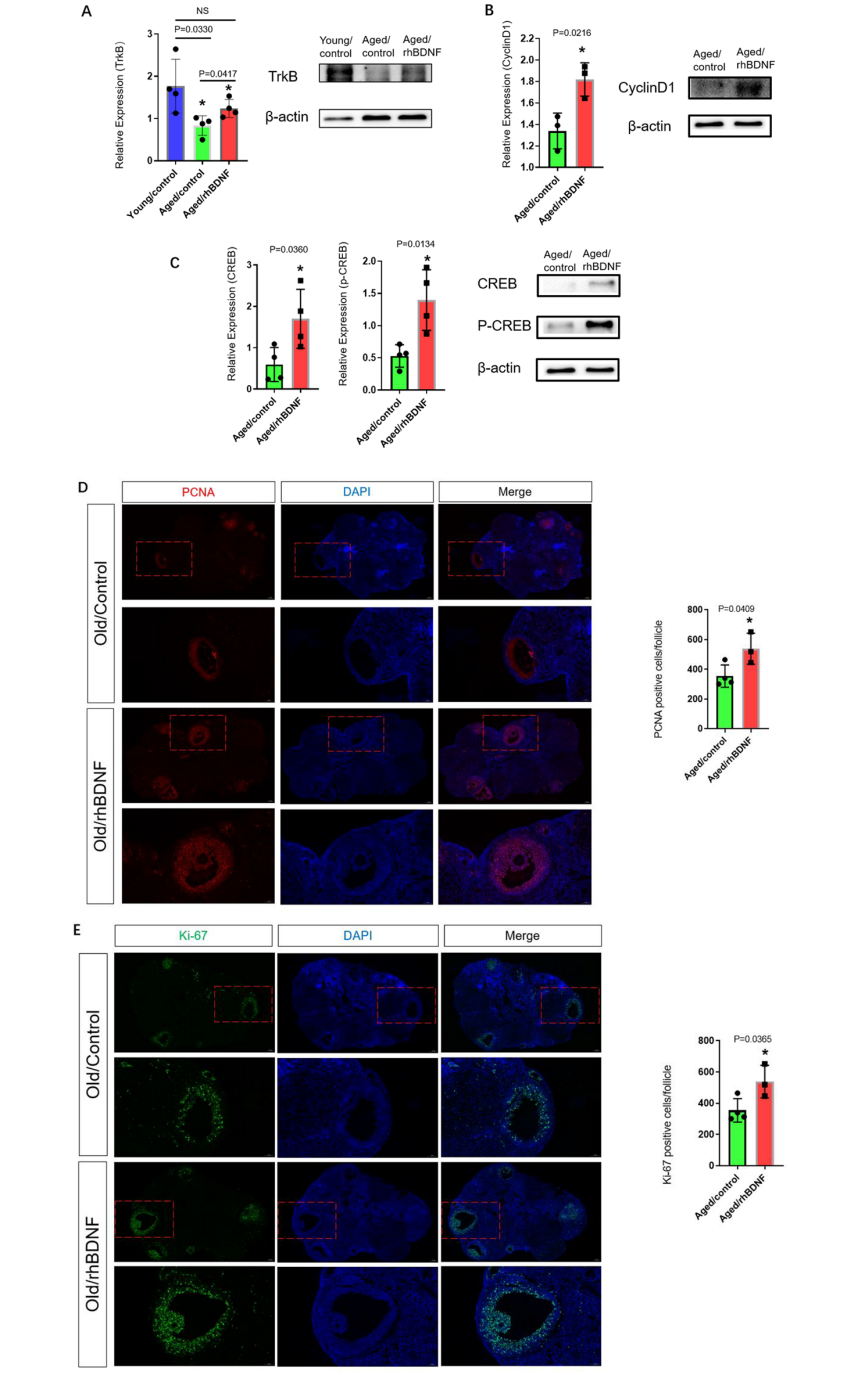
TrkB and Creb signaling and cell proliferation biomarkers in ovaries. (**A**) The densitometry of TrkB bands in Western blot analysis and a representative Western blot image. (**B**) The densitometry of cyclin D1 bands in Western blot analysis and a representative Western blot image. (**C**) The densitometry of creb and p-creb bands in Western blot analysis and a representative Western blot image. (n = 4) (**D**) Immunofluorescence staining of PCNA in ovaries. (**E**) Immunofluorescence staining of Ki-67 in ovaries. rhBDNF group: n = 3; control group: n = 4. Data are presented as mean ± SD. * indicates P < 0.05, ** indicates P < 0.01

TrkB protein expression was significantly decreased in ovaries of aged mice compared to ovaries of young mice. rhBDNF supplementation significantly increased TrkB protein expression in ovaries of aged mice compared to the aged mice without rhBDNF treatment (Fig. 6A). The protein levels of creb, phosphorylated-creb (p-creb), cyclin D1, and actin were measured using Western blot analysis. Creb binds to the cAMP response element (CRE) and plays key roles in cell survival, proliferation, and differentiation [40, 41]. Cyclin D1 is another key player for cell proliferation through regulation of the G1/S transition [42, 43]. The protein levels of proliferating cell nuclear antigen (PCNA) and Ki-67 were measured using immunofluorescence staining. PCNA, involved in DNA replication, DNA repair, and cell cycle control, is used as a marker for cell proliferation [44]. Ki-67 is another commonly used cell proliferation biomarker [45]. We demonstrated that the protein levels of creb, p-creb and cyclin D1 were significantly increased in ovaries collected from aged mice treated with rhBDNF compared to those in the control aged mice treated with normal saline (Fig. 6B-C). Immunofluorescence staining showed that the number of PCNA-positive ovarian cells and Ki-67-positive ovarian cells was significantly increased in the rhBDNF-treated group compared to the control group (Fig. 6D-E). No significant differences in the protein levels of bcl-2 and cleaved caspase-3, apoptosis biomarkers, were observed between the two groups (Fig. S4A and B). These results demonstrated that cell proliferation, but not apoptosis, signals were activated in rhBDNF-treated ovaries.

## Discussion

This study investigated the role of BDNF-TrkB signaling in ovarian aging. We demonstrated that ten consecutive days of daily IP injection of rhBDNF rescued ovarian function in aged mice, in part by increasing both the volume and mass of ovaries and the number of follicles. rhBDNF treatment also improved the number and quality of retrieved oocytes after ovulation induction in aged mice. The rate of fertilization, blastocysts formation (in the retrieved oocytes), and levels of estradiol (E2) were also higher following rhBDNF treatment. Ultimately, rhBDNF treatment increased the pregnancy rate in aged mice. Conversely, five consecutive days of daily IP injection of ANA 12, a TrkB antagonist, decreased both the volume and mass of ovaries and the number of antral follicles and increased the number of abnormal oocytes after ovulation induction in young mice. We further demonstrated that the role of BDNF-TrkB in ovarian function may be mediated by cyclin D1-creb signaling and cell proliferation.

The follicle is the key functional unit of female reproduction, and a reduction in the number of follicles is associated by a decline in ovarian function. The development of the follicle is a complex and precise process that is complemented by the maturation of oocytes and the proliferation and differentiation of surrounding granulosa cells. Granulosa cell proliferation predicts follicular development, and follicle atresia is triggered by apoptosis of granulosa cells [46]. Ovarian senescence has been considered an irreversible, natural phenomenon that leads to the decline of female reproductive function [47]. However, we showed that rhBDNF treatment activated ovarian cell proliferation and restored the function of aged ovaries in mice in our study.

The interaction between BDNF and TrkB promotes cell proliferation and inhibits apoptosis [48]. We found a positive correlation between the serum levels of BDNF and ovarian aging in mice. Our studies using ANA 12 treatment suggested that TrkB, one of the BDNF receptors that is expressed in oocytes and granulosa cells surrounding oocytes [49], may moderate follicle development in mice. These results are in concordance with previous studies about the role of BDNF-TrkB in the development of primordium follicles into antral follicles at all stages. BDNF-TrkB signaling promotes the formation of primary oocytes [50, 51]. In TrkB-deficient mice, granulosa cell proliferation, oocyte growth, and early follicular development were impaired [52]. BDNF-Trkb signaling supports early follicular development through a pathway driven by JAGGED1 production in oocytes [50]. The interaction between BDNF and TrkB promoted the proliferation of granulosa cells by the upregulation of growth differentiation factor 9 (GDF-9) or NOTCH2 receptor [50]. In the secondary oocyte stage, BDNF-TrkB upregulated the expression of follicle-stimulating hormone receptor (FSHR) and luteinizing hormone (LH) secretion, both of which can promote the proliferation of granulosa cells by activating the downstream signaling pathway [53]. In the antral follicle stage, BDNF-TrkB signaling promoted cell proliferation and inflammation through steroids and prostaglandins [54]. In our study, we discovered that the upregulation of p-Creb and PCNA in rhBDNF-treated aged ovaries might mediate BDNF-TrkB signaling. Creb, an essential transcription factor whose transcriptional effect is mediated by its phosphorylation [55, 56], has been shown to mediate this role of BDNF-TrkB signaling [57]. p-Creb could upregulate PCNA expression via promotion of its transcript process [58]. In addition, cyclin D1 is a key regulator of cell proliferation; the increase in cyclin D1 expression initiates the cell cycle from G1 phase to S phase, which promotes cell proliferation [59].

There is evidence to suggest that estrogen is a key regulator of circulating concentrations of BDNF [60]. In our study, our results suggest that BDNF may regulate circulating estrogen levels as well. Estrogen levels in postmenopausal women are significantly lower than those in women of reproductive age [61]. We showed that the estrogen levels in aged mice are also significantly lower than in young mice, which was in accordance with the decreased BDNF levels in these mice. Estradiol is mainly secreted by granulosa cells in ovaries. The synthesis of estrogen is a complex process: in granulosa cells, the phosphorylation of cAMP response element binding protein (CREB) leads to the activation of steroid hormone production such as the synthesis of estrogen, which is an important raw material for estradiol synthesis [62, 63]. Thus, rhBDNF treatment may increase estrogen levels by promoting the proliferation of granulosa cells.

BDNF is metabolized quickly in the body with a half-life < 1 min in plasma in rats [59, 64] and 3 h [65] with subcutaneous injection in rats. To maintain a continuous supply of BDNF in vivo, we chose a treatment regimen of daily injection of BDNF at a dose used in previous studies for 10 days to ensure the continuous supply of exogenous of rhBDNF. Intraperitoneal injection of rhBDNF at 1 µg/mouse can effectively improve the depression induced by chronic unpredictable stress [32, 33]. The strategy of intraperitoneal injection could be the quickest route for injected rhBDNF to reach to the ovaries [32, 33]. Under our BDNF treatment strategy, the pregnancy rate was not improved at the first cycle of mating (4th day of mating) but was improved until the second cycle of mating (8th day of mating). We performed a pilot study to test 5-day treatment of rhBDNF. The weight and size of ovaries did not significantly change with this shorter duration of treatment (Fig. S5). These data indicate that the dose and duration of BDNF treatment are essential for its efficacy in our study. Moreover, under our rhBDNF treatment strategy, we did not observe abnormal vital signs, such as body weight, in the mice. In addition, among pregnant mice, the numbers of embryo implantation sites in the rhBDNF-treated group compared to the control group were not different. Although there was only one pregnant mouse in the aged group due to fertility issues, our results suggest that improved reproductive function in rhBDNF-treated, aged mice were due to improved ovarian function and not changed in the uterus. As uterus aging has not been commonly observed in 35-37-week-old mice used in this study and is out of our study scope, we did not pursue this further.

ANA 12, a TrkB antagonist that blocks TrkB signaling by selectively binding to the extracellular domain of TrkB, is the first nonpeptide antagonist of TrkB receptor that elicits strong and specific effects in vivo [39]. Our treatment strategy was determined by referencing our pilot study and previous studies. Five days of ANA 12 (0.5 mg/kg) treatment has been shown to efficiently inhibit TrkB activity in mice [39, 66]. As inhibition of TrkB signaling by ANA 12 may inhibit neural signals [64, 67], studies have verified that ANA 12 (0.5 mg/kg) can be chronically administrated without toxic effects on the brain [67]. In our pilot study, ANA 12 (0.5 mg/kg) treatment by daily IP injection for 10 days resulted in death in some mice. Ovarian size and volume were not significantly different between mice intraperitoneally injected with ANA 12 (0.5 mg/kg) for 10 days vs. 5 days, so we chose the shorter duration as our treatment strategy. Previous studies indicated that BDNF-TrkB signaling is important for the development of primordium follicles into antral follicles at all stages. Although BDNF treatment increased the number of follicles in all stages in aged mice in our study, ANA 12 injection did not change the number of follicles at the primordial, primary, and secondary stages, but decreased the number of antral follicles and the quality of oocytes. These results could be due to the critical roles of TrkB in granulosa cells, which proliferate at a low level in early stages [52, 68, 69] but at a high level in later stages [70] of follicle development. In the secondary follicular stage, granulosa cells proliferate extensively from compact to loosen state, a transition that is critical for maturation of the follicles. We and others have shown that BDNF-TrkB signaling plays an important role of granulosa cell proliferation.

Our study has a limitation. The number of blastocysts obtained after IVF is low in our study. Blastocyst formation is related to the culture environment and the operation before IVF. In order to count the oocytes, we perform oocyte de-granulosa before we performed the IVF. We believe this is the reason that the number of blastocysts is low. Although the difference is small, we have observed this difference consistently under the same culture condition and environment.

## Conclusions

BDNF treatment significantly improved ovarian functions and reproduction in aged mice. Inhibition of BDNF-TrkB signaling resulted in impaired ovary function in young mice. The role of BDNF-TrkB signaling in the ovary may be mediated through creb and cell proliferation. Our results suggest that targeting BDNF-TrkB can be a novel therapeutic strategy for improving ovarian function in older women and young women with early ovary aging.

## Electronic supplementary material

Below is the link to the electronic supplementary material.

**Supplementary Material 1**

## Data Availability

All data generated or analysed during this study are included in this article. Raw data are available from the first and corresponding author upon reasonable request.

## List of abbreviations

BDNF: Brain-derived neurotrophic factor
rhBDNF: recombinant human BDNF protein
TrkB: Tropomyosin receptor kinase B
PMSG: Pregnant Mare Serum Gonadotrophin
Bcl2: B-cell lymphoma-2
E2: Estradiol
P4: Progesterone
PCNA: Proliferating cell nuclear antigen
FSHR: Follicle-stimulating hormone receptor
LH: Luteinizing Hormone
