# Supplementary material for: BDNF promotes mouse follicular development and reverses ovarian aging by promoting cell proliferation
Source: J Ovarian Res. 2023 Apr 27;16:83. doi: 10.1186/s13048-023-01163-9 (PMC10134588; doi:10.1186/s13048-023-01163-9)
Supplement: Supplementary file 1 — Supplementary Material 1 [file 13048_2023_1163_MOESM1_ESM.docx]

**[Supplementary material](http://dev.biologists.org/lookup/suppl/doi:10.1242/dev.081778/-/DC1)**


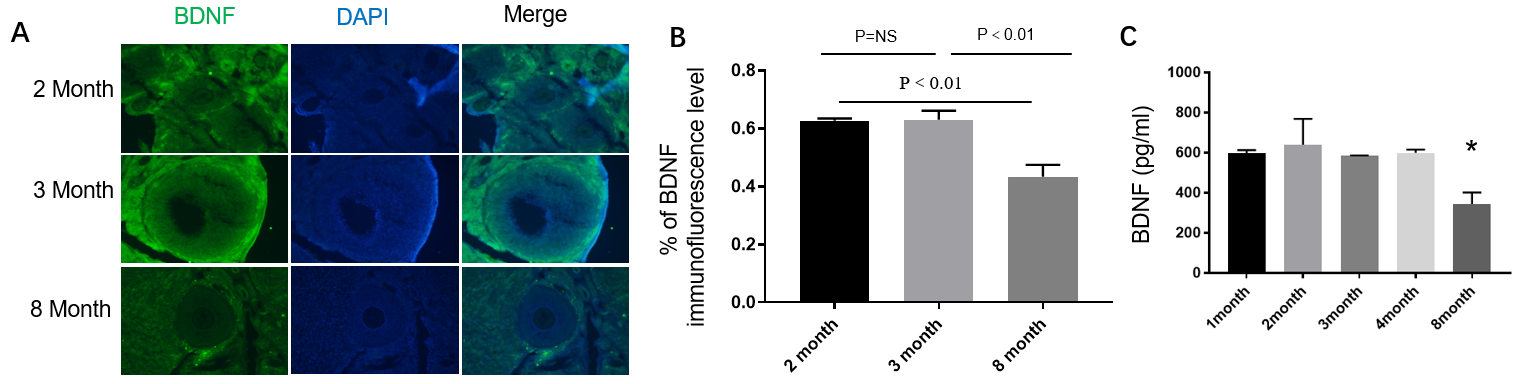


**Fig S1:** (A-B) The expression of BDNF in ovaries was detected by immunofluorescence. (C) The serum levels of BDNF were measured by ELISA. Data are shown as mean± SD. * indicates P < 0.05, ** indicates P < 0.01.


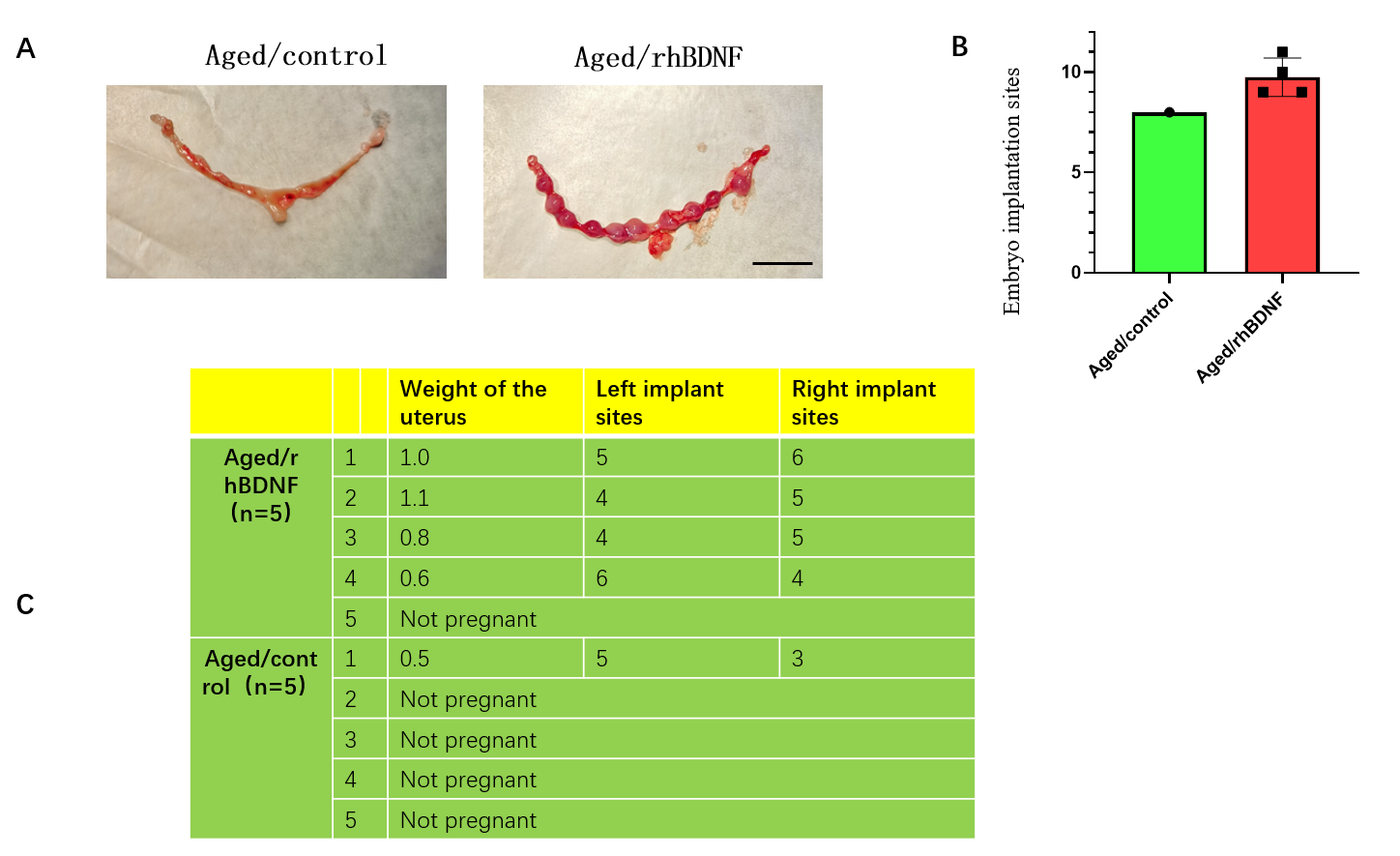


**Fig S2:** (A) Representative images of uterus on day 10.5–11.5. (B-C) Embryo implantation sites. Data are shown as mean± SD. * indicates P < 0.05, ** indicates P < 0.01.


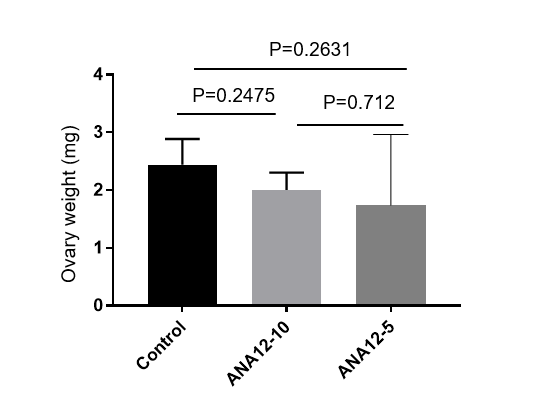


**Fig S3:** The ovarian weight (mg) of the control mice or mice treated with ANA 12 for 5 days or 10 days. Data are shown as mean± SD. * indicates P < 0.05, ** indicates P < 0.01.


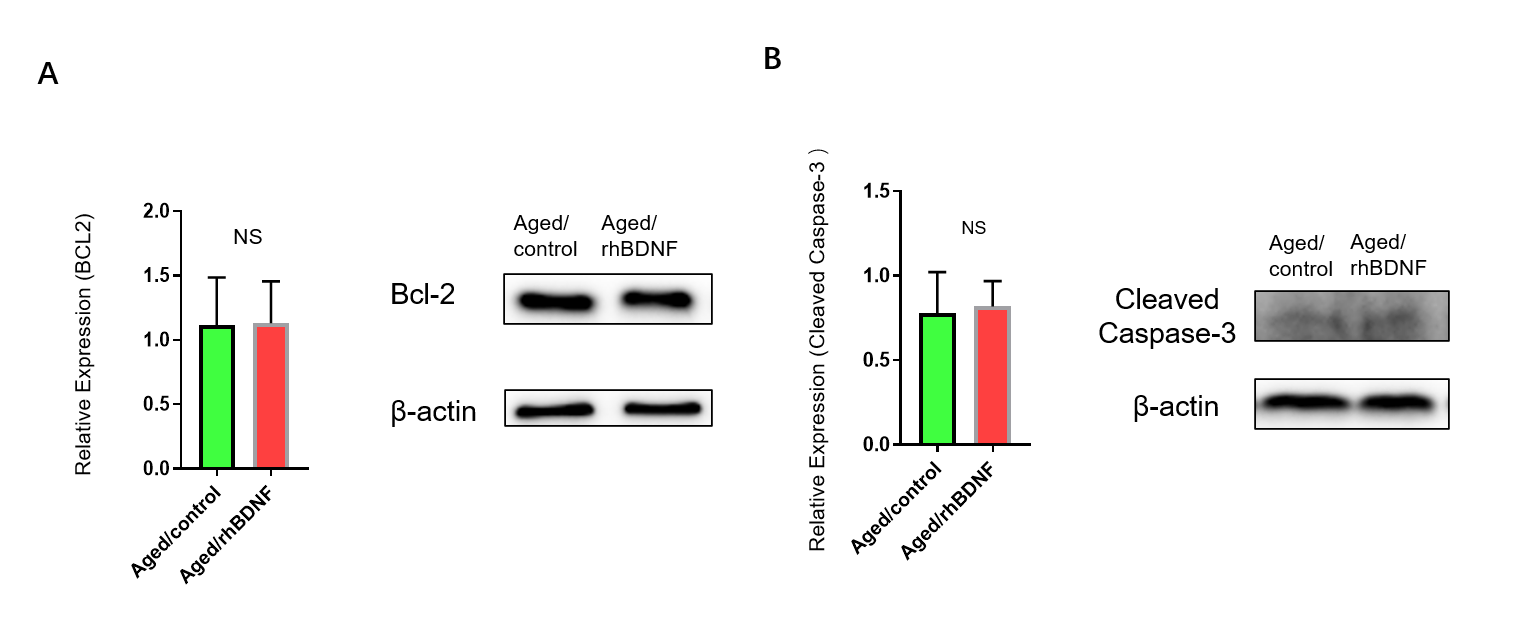


**Fig S4:** (A) The densitometry of Bcl2 bands in Western blot analysis and a representative Western blot image. (B) The densitometry of Cleaved Caspase-3 bands in Western blot analysis and a representative Western blot image.


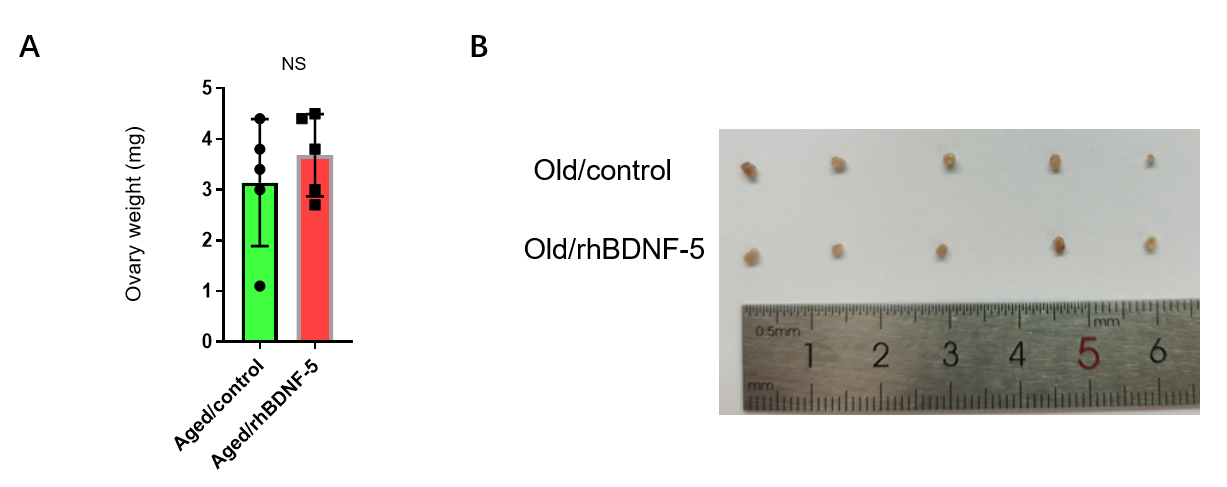


**Fig S5:** (A) Ovarian weight and (B) ovarian mass and volume in the control aged mice or aged mice with 5-day treatment of rhBDNF.
